# Transcriptome Sequencing of Two Phenotypic Mosaic *Eucalyptus* Trees Reveals Large Scale Transcriptome Re-Modelling

**DOI:** 10.1371/journal.pone.0123226

**Published:** 2015-05-15

**Authors:** Amanda Padovan, Hardip R. Patel, Aaron Chuah, Gavin A. Huttley, Sandra T. Krause, Jörg Degenhardt, William J. Foley, Carsten Külheim

**Affiliations:** 1 Research School of Biology, Australian National University, Canberra, ACT 0200, Australia; 2 Genome Discovery Unit, John Curtin School of Medical Research, Australian National University, Canberra, ACT 0200, Australia; 3 Institut für Pharmazie, Martin-Luther Universität Halle-Wittenberg, 06120, Halle (Saale), Germany; University of North Carolina at Charlotte, UNITED STATES

## Abstract

Phenotypic mosaic trees offer an ideal system for studying differential gene expression. We have investigated two mosaic eucalypt trees from two closely related species (*Eucalyptus melliodora* and *E*. *sideroxylon*), which each support two types of leaves: one part of the canopy is resistant to insect herbivory and the remaining leaves are susceptible. Driving this ecological distinction are differences in plant secondary metabolites. We used these phenotypic mosaics to investigate genome wide patterns of foliar gene expression with the aim of identifying patterns of differential gene expression and the somatic mutation(s) that lead to this phenotypic mosaicism. We sequenced the mRNA pool from leaves of the resistant and susceptible ecotypes from both mosaic eucalypts using the Illumina HiSeq 2000 platform. We found large differences in pathway regulation and gene expression between the ecotypes of each mosaic. The expression of the genes in the MVA and MEP pathways is reflected by variation in leaf chemistry, however this is not the case for the terpene synthases. Apart from the terpene biosynthetic pathway, there are several other metabolic pathways that are differentially regulated between the two ecotypes, suggesting there is much more phenotypic diversity than has been described. Despite the close relationship between the two species, they show large differences in the global patterns of gene and pathway regulation.

## Introduction

Differential gene expression presents species with a huge adaptive potential in an ever-changing world. There is evidence of this from across the tree of life. For example, antibiotic resistance in many strains of pathogenic bacteria, which is often the result of differential gene expression, is a continual problem for medicine. This means that some individual bacteria survive better in the presence of antibiotics than others [1]. Other examples are the expression of two genes that alter the ability of *Arabidopsis thaliana* plants to withstand different levels of drought stress [2], the differential expression of a single gene that determines lactose tolerance in non-infant humans [3] and the expression of 72 WRKY transcription factors changes in *Arabidopsis* plants as part of the defence response to biotic and abiotic stimuli [4].

There are many cases where differential gene expression is beneficial and can be used by a species to diversify its interaction with the environment [5]. There are two chemotypes of *Melaleuca quinquenervia* that differ in their expression of terpene synthases and this leads to differences in susceptibility to insect herbivores [6–8]. The converse of the situation is that differential gene expression may be deleterious to survival. For example, human cancers often result from changes to the expression patterns of just a few genes (*e*.*g*.: [9]). Although there are few examples, changes in gene expression may have no effect on an organism’s interaction with its environment: for example, many genes are differentially regulated in Desiree potatoes with no detectable phenotypic change [10].

Differential gene expression can be constitutively altered, as in the case of lactose tolerance [3] or the change may be induced under specific conditions, for example drought stress [2]. Whilst many studies have explored variation in gene expression as a result of changing environment [11–13], fewer studies have explored constitutive changes to gene expression within a population, with the prominent exception of human diseases. An example from the plant kingdom can be found with terpene biosynthesis genes in different *Melaleuca alternifolia* chemotypes which are constitutively differentially expressed [14]. Most studies on differential gene expression examine particular candidate genes in response to a known environmental change [15–17], or compare genome-wide expression patterns of different species in the same environment in search of the evolutionary patterns [18–20].

Plants provide a unique environment to investigate differential gene expression since they are made up of a series of clones (or ramets) each with their own stem cells (meristems) [21], such that a permanent change in the regulation of gene expression could lead to an entire branch, including leaves, stems, flowers, seeds and pollen with different gene expression compared to the rest of the plant. The genetic mosaic hypothesis [21–22] was proposed to explain how asexual organisms evolve. There are many horticultural examples that highlight the importance of mosaicism for sexual organisms as well. For example the nectarine is a genetic variant of the peach and both originally co-occurred on the same tree [23]. Unlike in animal systems, somatic mutations in plants can be passed onto the next generation since meristems give rise to all tissues on a branch, including reproductive tissue [24], and thus there is the potential for genetic mosaicism to confer an adaptive advantage.

**Fig 1:**
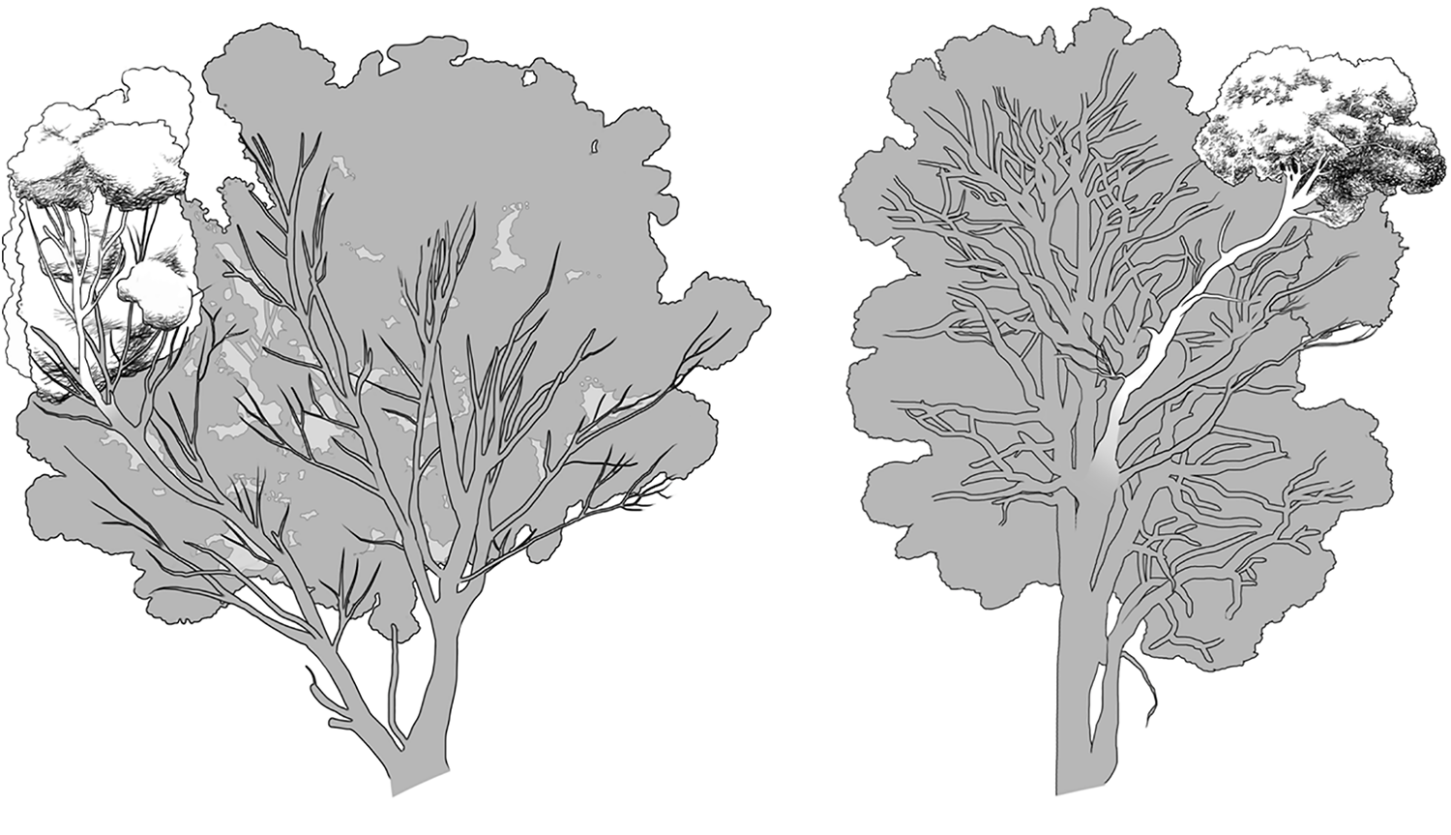
A schematic of the *E*. *melliodora* (left) and the *E*. *sideroxylon* (right) mosaic trees. The white areas in the canopy represent the leaves resistant to herbivory and the grey areas represent leaves that are susceptible to herbivory (graphic by Erin Walsh 2012).

Here we report genome-wide transcript abundance of the same tissue in the same species and individual, with known striking phenotypic differences in both herbivore resistance and plant secondary metabolites. Two individuals from different species, one *Eucalyptus melliodora* and one *E*. *sideroxylon* (Fig 1) were investigated. Most of the leaves in each tree have a terpene and formylated phloroglucinol compound (FPC) profile that makes them susceptible to insect and mammalian herbivory, whereas leaves on one branch have a different terpene and FPC profile that confers resistance to these herbivores (Fig 1) [25–28]. This is a particularly interesting system, since the chemical defences are constitutive and not induced by herbivory, therefore all genetic differences between the branches must be constitutive. We investigated genome-wide differences in foliar gene expression and use the genes involved in the terpene biosynthetic pathway to show whether differences in gene expression correlate with phenotypic variation.

The motivation for this work is to identify the somatic mutation that resulted in two distinct phenotypes occurring in the leaves within one tree. Towards this objective we have three specific aims, to:

1. Compare the patterns of gene expression between resistant and susceptible leaves of the same tree and the same chemotype between trees, including expression of genes and of entire biosynthetic pathways
2. Determine if the patterns of gene expression correlate with phenotype
3. Predict genes that could influence and control the phenotypic mosaicism in each species

We have previously used similar methods to investigate gene expression within one mosaic *(E*. *melliodora*) [29]. Here, we include multiple samples per chemotype, use more advanced technology to generate the sequence data and a more robust pipeline to analyse this data, as well as including a second species with a similar mosaic phenotype. This study is a significant advancement on the previous study since the data allowed us to identify specific genes that are differentially expressed and may be involved in the development and maintenance of phenotypic mosaicism in these trees.

## Results and Discussion

Sequencing pools of mRNA from leaves of resistant and susceptible branches from two individuals of eucalypts, we obtained 193,877,527 reads from 10 samples (Table 1, Biosample Project SAMN03015957, SAMN03015958, SAMN03015959, SAMN03015960, SAMN03015961, SAMN03015962) and have used these data to address our aims.

**Table 1:** RNASeq sequencing statistics.

Sample	Tag Counts	Mapped Tags
S1R2	8 753 290	2 301 804
S1S1	19 314 186	5 546 899
S1S2	11 577 830	3 240 695
S1S5	12 675 035	3 257 847
MHR1	24 316 848	6 638 586
MHR2	24 333 048	7 642 163
MHR3	21 281 113	5 131 033
MHS1	16 383 774	4 572 200
MHS3	13 046 415	3 885 764
MHS4	42 195 988	12 068 462

The tag counts and number of mapped tags for RNASeq of 10 samples from two phenotypically mosaic eucalyptus trees. *Eucalyptus sideroxylon* is denoted by S1 and *E*. *melliodora* is denoted by MH. In both species R refers to leaves resistant to herbivory and S refers to leaves susceptible to herbivory, the final number indicates the branch number.

### How does gene expression vary between the resistant and susceptible leaves within a mosaic tree?

We used a gene set enrichment analysis to compare the gene expression between the leaves of the two chemotypes in each mosaic (Table 2). There is no significant difference between the gene sets expressed in the resistant and susceptible leaves of the *E*. *sideroxylon* mosaic (Wilcoxon matched pairs test *p>*0.05—Table 2). However, there is a significant difference between the genes expressed in the leaves of the *E*. *melliodora* mosaic (Wilcoxon matched pairs test *p*<0.05—Table 2). Having said that, there are 1646 and 1549 genes differentially expressed between the resistant and susceptible chemotypes of the *E*. *melliodora* and *E*. *sideroxylon*, respectively.

**Table 2:** Gene set enrichment analysis.

*Eucalyptus sideroxylon*			*Eucalyptus melliodora*		
GO Term (Biological process)	S1S	S1R	GO Term (Biological processes)	MHS	MHR
other cellular processes	0.30	0.28	other cellular processes	0.09	0.20
other metabolic processes	0.23	0.20	other metabolic processes	0.09	0.19
response to stress	0.07	0.11	response to stress	0.04	0.12
response to abiotic or biotic stimulus	0.08	0.09	response to abiotic or biotic stimulus	0.04	0.10
transport	0.06	0.07	other biological processes	0.02	0.09
other biological processes	0.05	0.06	transport	0.03	0.07
signal transduction	0.03	0.04	developmental processes	0.03	0.04
protein metabolism	0.04	0.04	cell organization and biogenesis	0.02	0.04
developmental processes	0.05	0.03	protein metabolism	0.03	0.04
cell organization and biogenesis	0.04	0.03	unknown biological processes	0.02	0.04
unknown biological processes	0.02	0.02	signal transduction	0.02	0.03
Transcription, DNA-dependent	0.02	0.01	transcription, DNA-dependent	0.02	0.02
electron transport or energy pathways	0.01	0.00	electron transport or energy pathways	0.01	0.02
DNA or RNA metabolism	0.01	0.00	DNA or RNA metabolism	0.00	0.00
Wilcoxon matched pairs test		*p* = 1.00	Wilcoxon matched pairs test		*p<*0.001
**GO Term (Molecular function)**	**S1S**	**S1R**	**GO Term (Molecular function)**	**MHS**	**MHR**
transferase activity	0.17	0.18	other enzyme activity	0.08	0.19
other binding	0.17	0.15	other binding	0.12	0.18
other enzyme activity	0.13	0.14	nucleotide binding	0.06	0.10
transporter activity	0.06	0.11	transferase activity	0.05	0.10
kinase activity	0.10	0.10	unknown molecular functions	0.07	0.08
hydrolase activity	0.04	0.09	protein binding	0.05	0.08
nucleotide binding	0.08	0.07	hydrolase activity	0.04	0.08
unknown molecular functions	0.04	0.05	transporter activity	0.02	0.07
protein binding	0.07	0.04	other molecular functions	0.03	0.05
DNA or RNA binding	0.04	0.03	DNA or RNA binding	0.05	0.02
transcription factor activity	0.03	0.02	transcription factor activity	0.04	0.02
other molecular functions	0.03	0.02	kinase activity	0.03	0.02
nucleic acid binding	0.01	0.01	structural molecule activity	0.01	0.01
receptor binding or activity	0.00	0.01	receptor binding or activity	0.00	0.01
structural molecule activity	0.01	0.00	nucleic acid binding	0.01	0.00
Wilcoxon matched pairs test		*p* = 0.60	Wilcoxon matched pairs test		*p* = 0.03

The left side of this table contains the data for the *E*. *sideroxylon* mosaic and the right side contains the data for the *E*. *melliodora* mosaic. The GO term or category is listed in the first column of each side, with the biological processes GO terms followed by the molecular function GO terms. The numbers in the final two columns on each side show the proportion of transcripts found for each GO term in the leaves of the susceptible branch (S1S and MHS) and those of the resistant branch (S1R and MHR).

These results show that the two mosaic trees, of different species, have different global patterns of gene expression. Unless the mutation in each mosaic tree affected exactly the same nucleotide of the same gene, then it is reasonable to expect the phenotype (and associated genetics) to be different. Indeed there are subtle differences in the terpene and FPC profile of the resistant leaves of the *E*. *melliodora* mosaic compared with those of the *E*. *sideroxylon* mosaic (the same is true for the susceptible branches of each mosaic tree) [28]. Since the two species show different patterns of gene expression, we will treat them as separate studies by presenting and discussing the results of each independently, followed by a short comparison of the two mosaic trees at the end.

The differences in gene expression within the *E*. *melliodora* mosaic is likely driven by the large differences in the expression of genes involved in the biological functions of 'response to stress' (proportion of transcripts in this GO category in each mosaic, *E*. *melliodora* resistant leaves (MR): *E*. *melliodora* susceptible leaves (MS) 0.12:0.04) and 'response to biotic or abiotic stimuli' (MHR:MHS 0.10:0.04) as well as those involved in the molecular functions of 'nucleotide binding' (MHR:MHS 0.10:0.06) and 'transferase activity' (MHR:MHS 0.10:0.05) (Table 2), all of which are over-represented in the leaves of the resistant branch of the *E*. *melliodora* mosaic. These gene expression differences can explain the terpene and FPC variation described for both mosaic trees, however, we can determine the specific biosynthetic pathways whose genes are contributing to the over-represented gene ontology categories.

**Fig 2:**
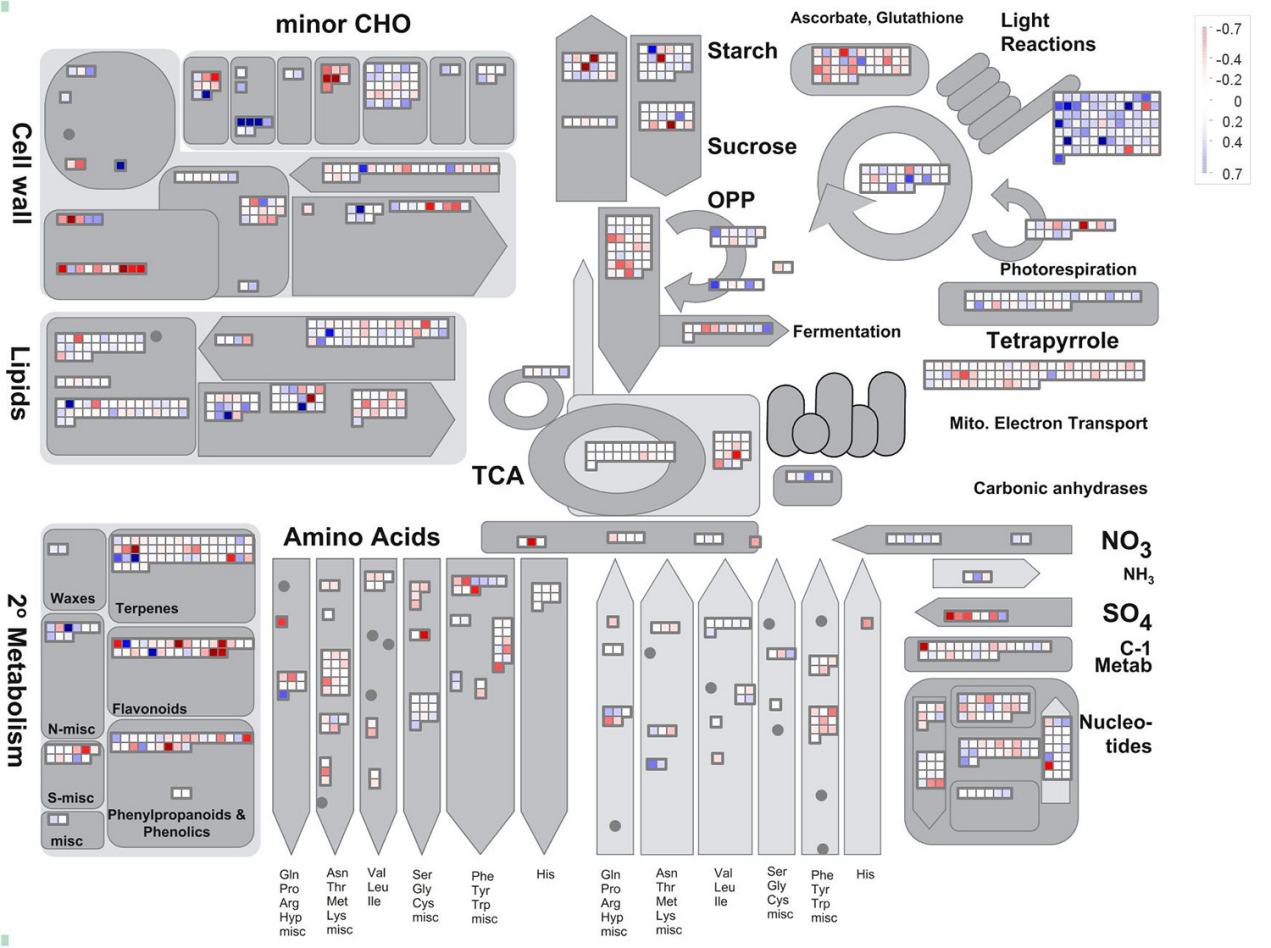
Pathway analysis of genes expressed in the E. melliodora mosaic. Blue squares indicate transcripts with higher levels of abundance in the leaves of the susceptible branch and red squares indicate transcripts more abundant in the leaves of the resistant branch. The intensity of the colour indicates the level of differential abundance of a transcript.

Each enzyme within a biosynthetic pathway is dependent on all of the other enzymes in this pathway, and thus we expect there to be a co-ordinated regulation of the gene expression and protein action of each [14]. Therefore, individual genes within the pathway may not be differentially expressed and would not be picked up in traditional gene expression analyses, but rather the group of genes (biosynthetic pathway) is up or down regulated under a particular treatment. This has been shown in *Arabidopsis thaliana*, where Frenkel *et al*. [30] found the photosynthetic pathway in two mutant lines and corresponding wild type was significantly up-regulated in one *Arabidopsis* line, but found no evidence of individual genes being differentially expressed between the lines. Here we use a similar technique to identify biosynthetic pathways that are differentially regulated between the leaves of the resistant and susceptible chemotypes in each mosaic (Table 2, Fig 2).

In the *E*. *melliodora* mosaic, transcripts associated with light reactions (GO: response to abiotic stimuli) are consistently over-represented in the leaves of the resistant branch, as well as those associated with oxidation of pyrophosphate groups (GO: response to biotic and abiotic stimuli) and trehalose metabolism (GO: response to abiotic stimuli). In the leaves of the susceptible branch of the same species, transcripts associated with Krebs cycle (TCA; GO: response to biotic and abiotic stimuli), sulphur assimilation (GO: other cellular process) and modifying the cell wall (GO: cell organisation and biogenesis) are over-represented (Fig 2). These results suggest that the leaves of the resistant branch are devoting more resources to a response to abiotic and/or biotic stimuli than those of the susceptible branch. Conversely the leaves of the susceptible branch are undergoing more cellular processes, which could be attributed to outgrowing herbivore-induced damage. These patterns are consistent with the GO analysis, as many of the pathways over-represented in the resistant leaves are involved in the plants response to biotic and abiotic stimuli [29].

**Fig 3:**
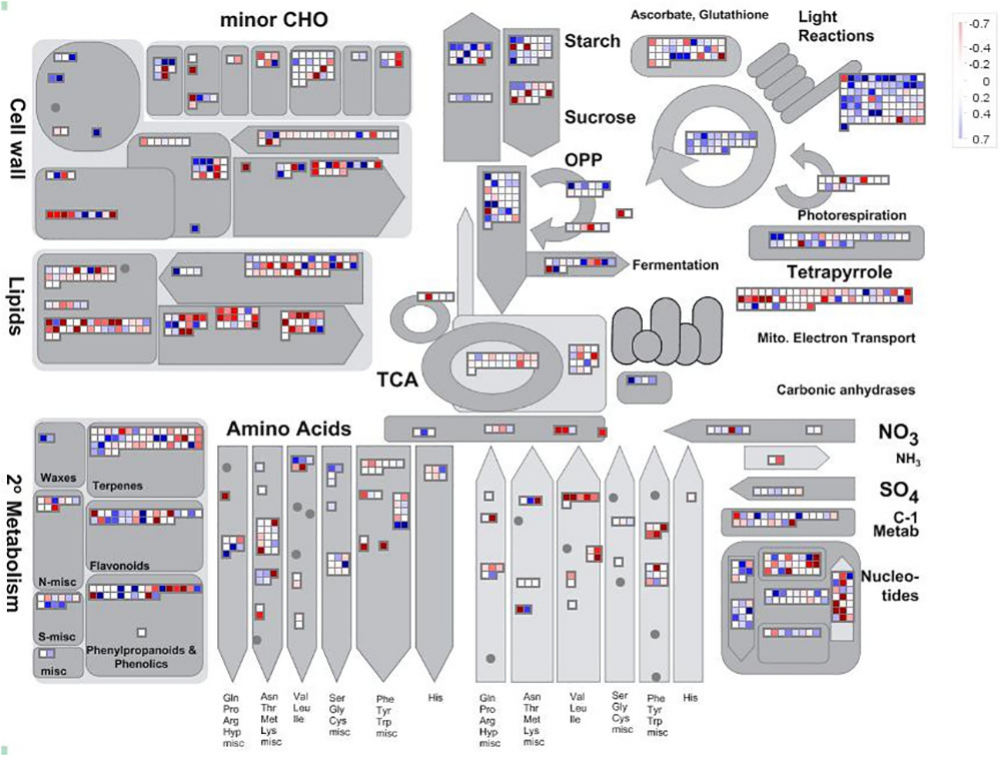
Pathway analysis of genes expressed in the E. sideroxylon mosaic. Blue squares indicate transcripts with higher levels of abundant in the leaves of the susceptible branch and red squares indicate transcripts more abundant in the leaves of the resistant branch. THE intensity of the colour indicates the level of differential abundance of a transcript.

Despite individual genes not being differentially expressed within the *E*. *sideroxylon* mosaic, we did find evidence of entire pathways being differentially regulated. Transcripts associated with light reactions (GO: response to abiotic stimulus), the Calvin Cycle (GO: response to abiotic and biotic stimulus), cell wall protein synthesis (GO: cell organisation and biogenesis), wax synthesis (GO: other metabolic process), the transformation of carbonic anhydrides (GO: other metabolic process) and the oxidation of pyrophosphate-containing compounds (GO: response to abiotic and biotic stimulus) are more abundant in the leaves of the resistant branch. The leaves of the susceptible branch contain an over-abundance of transcripts involved in the biosynthesis of many amino acids (GO: protein metabolism), lipid metabolism (GO: other metabolic process) and nucleotide degradation (GO: DNA or RNA metabolism—Fig 3). These results suggest that the leaves of the susceptible branch are investing resources in a response to abiotic and/or biotic stimuli as well as cellular processes that could be associated with damage repair. The leaves of the susceptible branch, however, are devoting resources to metabolic processes associated with gene expression, which could be attributed to the fact that there are many more genes expressed in the leaves of the susceptible branch compared with those of the resistant branch (Table 3).

**Table 3:** Significantly up regulated pathways in the leaves of the resistant branch (resistant) and the susceptible branch (susceptible) of each mosaic.

	*E*. *melliodora*	*E*. *sideroxylon*
**Resistant**	• light reactions	• light reactions
	• oxidation of pyrophosphate containing compounds	• Calvin cycle
	• trehalose metabolism	• cell wall protein synthesis
		• wax synthesis
		• transformation of carbonic anhydrides
		• oxidation of pyrophosphate containing compounds
**Susceptible**	• Krebs cycle (TCA)	• amino acid biosynthesis
	• sulphur assimilation	• lipid metabolism
	• modification of cell wall	• nucleotide degradation

Broad-scale transcriptomic analysis is rarely accompanied by functional characterization of specific genes to determine the degree to which gene expression and phenotype are linked. Given the central role of foliar terpenes in the mosaic phenotype we aimed to determine the function of some of the most highly expressed terpene synthases in both the resistant and susceptible leaves.

### Do the gene expression patterns correlate with phenotypic variation?

There is clear and documented variation in the terpene profile of the leaves from both chemotypes of the two mosaics [25–26, 28] and the genes involved in terpene biosynthesis are well known in many species, including eucalypts [31–34]. This enables us to compare these data to the expression levels of terpene biosynthetic genes to predict whether regulation of gene expression is sufficient to account for the terpene variation in these mosaic eucalypts.

Briefly, the leaves of the resistant branch have significantly higher concentrations of the monoterpene 1,8-cineole and lower concentrations of sesquiterpenes in both mosaic trees [28]. The *E*. *sideroxylon* mosaic contains more terpenes in the leaves of each branch when compared to the *E*. *melliodora* mosaic [28]. From this data we make three predictions: 1. That the genes in the MEP pathway will be over-represented in the leaves of the resistant branch in both species, compared to the leaves of the susceptible branch, 2. That the genes in the MVA pathway will be over-represented in the leaves of the susceptible branch in both species, compared to the leaves of the resistant branch, 3. There will be some terpene synthases that are over-represented in the leaves of the resistant branch (*e*.*g*. 1,8-cineole synthase) and others that are over-represented in the leaves of the susceptible branch (*e*.*g*. α-phellandrene synthase).

There are 17 terpene synthases that are differentially expressed between the two branches of each of the two mosaic trees. Of these, 10 are differentially expressed in both species and show the same pattern of regulation (*i*.*e*. up-regulated in the leaves of the same chemotype of the mosaic in both species). In the *E*. *melliodora* mosaic, there are four monoterpene synthases and 13 sesquiterpene synthases differentially expressed between the two branches (Table 4), whilst in the *E*. *sideroxylon* mosaic there is only one monoterpene synthase and 16 sesquiterpene synthases differentially expressed between the two branches (Table 5). Of the seven unique differentially expressed terpene synthases in each species, only one sesquiterpene synthase is up-regulated in the resistant branch. There are three other genes involved in the terpene biosynthetic pathway that are differentially expressed between the two chemotypes: *dxs2* is up-regulated in the leaves of the susceptible branch in the *E*. *melliodora* mosaic and the leaves of the resistant branch in the *E*. *sideroxylon* mosaic; *hmgr5* is up-regulated in the leaves of the susceptible branch of the *E*. *sideroxylon* mosaic and *hmgr3* is up-regulated in the leaves of the susceptible branch of the *E*. *melliodora* mosaic.

**Table 4:** Terpene biosynthetic genes differentially expressed between the resistant and susceptible leave of E. melliodora.

Gene ID	Phytozome ID	logFC	*p-*value	TPS-Fam
dxs2	Eucgr.I00566	-1.04	0.04	-
EgTPS009	Eucgr.C04198	-0.88	0.03	a
EgTPS016	Eucgr.E00404	4.01	0.02	a
EgTPS018	Eucgr.E00415	6.02	0.02	a
EgTPS019	Eucgr.E00419	6.19	0.01	a
EgTPS020	Eucgr.E02670	1.96	0.03	a
EgTPS021	Eucgr.E03115	-1.53	0.00	a
EgTPS025	Eucgr.F03398	2.21	0.01	a
EgTPS026	Eucgr.F03400	1.96	0.05	a
EgTPS027	Eucgr.F03400	1.96	0.05	a
EgTPS033	Eucgr.F03413	-2.03	0.00	a
EgTPS046	Eucgr.L02418	-1.53	0.00	a
EgTPS049	Eucgr.L02971	2.09	0.01	a
EgTPS051	Eucgr.L03307	-1.36	0.00	a
EgTPS078	Eucgr.K00828	1.35	0.00	b
EgTPS101	Eucgr.E03562	3.14	0.00	g
EgTPS102	Eucgr.E03563	3.43	0.01	g
EgTPS112	Eucgr.E03610	3.45	0.00	g
hmgr3	Eucgr.I01544	-1.00	0.01	-

The Gene ID column contains the name of each gene based on the closest match in the *E*. *grandis* genome (Külheim et al. 2013). The Phytozome ID column contains the gene identifier of the best match to the *E*. *grandis* genome found at http://phytozome.net/search.php?method=org_egrandis (Joint-Genome-Institute 2006–2013). The logFC column contains the log fold change data for each gene, where a negative log fold change value indicates the transcript is more abundant in the leaves of the susceptible branch than those of the resistant branch. The *p*-value column contains the *p*-value associated with the logFC for each gene. All of these values are <0.05 indicating the genes are significantly differentially expressed. The TPS-Fam column shows which terpene synthase subfamily the terpene synthases belong to (Külheim *et al*. 2013).

**Table 5:** Terpene biosynthetic genes differentially expressed between the resistant and susceptible leave of E. sideroxylon.

Gene ID	Phytozome ID	logFC	*p-*value	TPS-Fam
dxs2	Eucgr.I00566	1.32	0.01	-
EgTPS005	Eucgr.C02467	2.01	0.00	a
EgTPS006	Eucgr.C02474	1.80	0.01	a
EgTPS007	Eucgr.C02554	1.96	0.00	a
EgTPS020	Eucgr.E02670	8.42	0.01	a
EgTPS021	Eucgr.E03115	-1.09	0.00	a
EgTPS022	Eucgr.E03311	7.81	0.00	a
EgTPS025	Eucgr.F03398	4.48	0.03	a
EgTPS026	Eucgr.F03400	4.38	0.03	a
EgTPS027	Eucgr.F03400	4.38	0.03	a
EgTPS033	Eucgr.F03413	-1.21	0.00	a
EgTPS034	Eucgr.G01636	-5.20	0.05	a
EgTPS038	Eucgr.J01451	7.40	0.04	a
EgTPS041	Eucgr.K03518	5.26	0.03	a
EgTPS046	Eucgr.L02418	-0.89	0.04	a
EgTPS049	Eucgr.L02971	5.83	0.02	a
EgTPS051	Eucgr.L03307	-0.80	0.01	a
EgTPS071	Eucgr.D00872	3.47	0.04	b
hmgr5	Eucgr.I02200	-4.82	0.01	-

The Gene ID column contains the name of each gene based on the closest match in the *E*. *grandis* genome (Külheim et al. 2013). The Phytozome ID column contains the catalog number of the best match to the *E*. *grandis* genome found at http://phytozome.net/search.php?method=org_egrandis (Joint-Genome-Institute 2006–2013). The logFC column contains the log fold change data for each gene, where a negative log fold change value indicates the transcript is more abundant in the leaves of the susceptible branch than those of the resistant branch. The *p*-value column contains the *p*-value associated with the logFC for each gene. All of these values are <0.05 indicating the genes are significantly differentially expressed. The TPS-Fam column shows which terpene synthase subfamily the terpene synthases belong to (Külheim *et al*. 2013).

There is only one gene from each of the MVA and MEP pathways that is differentially expressed between the leaves of the two branches in each mosaic, which is accompanied by quantitative differences in both the monoterpenes and sesquiterpenes in each branch. This suggests that these genes, *dxs* and *hmgr*, could act as rate-limiting steps in the MEP and MVA pathways, respectively. Studies in transgenic *Arabidopsis thaliana* plants [35] showed *dxs* to be the rate-limiting step in the MEP pathway. However, two recent studies in natural populations of Myrtaceae have shown that the expression of *mcs* (the fifth step in the MEP pathway) and SNPs in *hdr* and *hds* (the two last steps in the MEP pathway) are significantly correlated with monoterpene yield in *Melaleuca alternifolia* [14] and *Eucalyptus globulus* [36], respectively. This needs to be investigated further, however, *dxs* appears to be a rate-limiting step in terpene biosynthesis within *E*. *melliodora* and *E*. *sideroxylon*, which is similar to what is shown in *Arabidopsis* but not in other Myrtaceae.

There are many more sesquiterpene synthases differentially regulated between the leaves of the resistant and susceptible branches within each mosaic tree. The sesquiterpene profile of the leaves of each branch is qualitatively the same, however the susceptible branch contains a significantly higher concentration of all sesquiterpenes than the resistant branch. Therefore, we would expect all sesquiterpene synthases (as well as the MVA pathway genes) to be over-represented in the susceptible leaves. Instead we see just 12 and 15 (of the 52 TPS-a (angiosperm sesquiterpene synthases) identified in the *E*. *grandis* genome) [34] over-represented in the susceptible branch of the *E*. *melliodora* and *E*. *sideroxylon* mosaics, respectively. A recent analysis of the terpene synthase gene family of apple (*Malus domestica*) suggests that of the 55 genes present in the genome, only 10 are functional and contributing to the terpene profile of the fruits [37]. Therefore, the 12 and 15 TPS-a genes over-represented in the leaves of the susceptible branch of each mosaic could easily account for the increased sesquiterpene concentration in the leaves of this branch.

It is noteworthy that few (five out of 17 in each mosaic tree) differentially expressed terpene synthases are putative monoterpene synthases (TPS-b (angiosperm monoterpene synthases), when compared to the *E*. *grandis* genome), and that they are all up-regulated in the leaves of the susceptible branch of the two mosaics. However, the phenotype data showed that most of the individual monoterpenes are found in higher concentrations in the leaves of the susceptible branch in the two mosaic trees [28]. A possible explanation for this could be that some of these terpene synthases are not contributing to the terpenes found within the secretory cavities (measured by [28]). Alternative explanations could be either post-transcriptional regulation or the appropriate substrates aren't in the correct cellular compartments.

Understanding the function of some of the differentially expressed terpene synthase genes provides further information to these arguments (Table 6). We characterised the function of six terpene synthases, all of which are highly expressed in at least one sample and three of which are differentially expressed between the leaves of the two chemotypes. All of the characterized genes fall into the TPS-a sub-family and are therefore putative sesquiterpene synthases. Three of these are β-caryophyllene synthases, two are germacrene D synthases and one is inactive. The five active enzymes are multi-product enzymes but only farnesyl pyrophosphate was utilised as a substrate. Both β-caryophyllene and germacrene D synthases show terpene profiles similar to those identified in other species [38–41].

**Table 6:** Functional characterization of terpene synthases.

*E*. *grandis* homolog		Gene	expression		Function
	EUsid	*p*-value	EUmel	*p*-value	
EgTPS05	-1.09	0.00	-1.53	0.12	β-caryophyllene
EgTPS21	-1.09	0.00	-1.53	0.00	inactive
EgTPS26	4.38	0.03	1.96	0.05	β-caryophyllene
EgTPS33	-1.21	0.00	-2.03	0.00	β-caryophyllene
EgTPS38	7.40	0.04	-1.02	0.22	germacrene D
EgTPS40	5.88	0.22	-0.81	0.48	germacrene D

Terpene synthases were amplified from leaves of the resistant and susceptible branches of two mosaic eucalypts. The first column contains the name of the *E*. *grandis* terpene synthase that is the closet match to the sequence we amplified. The next four columns contain the gene expression values with estimated significance levels from the transcriptome sequencing of the two mosaics. The two species are treated separately here. There is a log fold change and a *p*-value for each terpene synthase in the two mosaics (*E*. *melliodora* = EUmel; *E*. *sideroxylon* = EUsid). A negative log fold change value indicates the terpene synthase is more abundant in the leaves of the susceptible branch of that tree. The final column shows the most abundant terpene produced by the enzyme when artificially over-expressed in *E*. *coli*. All genes were incubated with GPP (the substrate for monoterpene synthases) and FPP (the substrate for sesquiterpene synthases) independently.

Whilst β-caryophyllene is found in both resistant and susceptible leaves of each mosaic, it is found in significantly higher concentrations in the susceptible leaves (R = 0.1672 mg∙g^-1^ DW, S = 3.2502 mg∙g^-1^ DW, p<0.01) and consistently ranks as one of the five most abundant terpenes in every susceptible leaf sample (data not shown). Interestingly the β-caryophyllene synthase that appears to produce the greatest amount of β-caryophyllene (EgTPS26) is up-regulated in the leaves of the resistant branch of the two mosaics whilst the β-caryophyllene synthase that produces the smallest amount of β-caryophyllene is up-regulated in the leaves of the susceptible branch of the two mosaics (EgTPS33). This suggests that in the resistant branch, β-caryophyllene may be further metabolised within the leaf or it may be released to the environment.

Germacrene D was not detected in the foliar terpene profile of either chemotypes of the two mosaics [28]. If a germacrene D synthase is expressed in the leaf and there is differential expression of this gene between the two ecotypes (resistant and susceptible leaves) then germacrene D may be playing a role in the resistance or susceptibility to the herbivory in a manner yet to be determined, possibly through further modification of the compound.

Zini et al., [42] showed that several compounds, including germacrene D and β-caryophyllene, can be found in the headspace of leaves of *E*. *citriodora*, *E*. *dunnii* and *E*. *saligna*, but not in the constitutive leaf oils of the same three species of *Eucalyptus*. The authors suggested that these differences illustrate something about the “infochemical role of these compounds in *Eucalyptus*,” but failed to elaborate. We hypothesise that there are two pools of foliar terpenes. One that is produced by the actions of TPS genes in the secretory cavities and stored there [43] and a second that is synthesised by terpene synthases distributed in other tissues (e.g. the palisade mesophyll) and emitted to the environment, where they influence tritrophic interactions. Preliminary studies from two different directions (isolated *Eucalyptus* secretory cavities (J Goodger and I Woodrow personal communication)) and ^13^C labelling experiments in eucalypts (A Winters personal communication) support this view.

In summary, some genes show the predicted expression patterns (based on phenotypic variation) however there are several inconsistencies. This is particularly evident in the terpene synthases where functional characterisation revealed enzyme products which are not found in the leaf oil profile [28]. Resolving the origins and locations of *Eucalyptus* terpene synthases is a priority.

### What genes could influence and control phenotypic mosaicism in these two species?

The motivation for doing this work was to identify the somatic mutation that resulted in two distinct phenotypes occurring in the leaves within one tree. In these mosaics, there is variation in at least three biosynthetically distinct groups of compounds, the monoterpenes, sesquiterpenes and FPCs and the biosynthesis of these compounds was shown to be co-ordinated in the leaves of the closely related species *E*. *nitens* [44]. We believe the somatic mutation is a single mutation because it has to have occurred in the meristem of a developing shoot. This leads us to the hypothesis that the somatic mutation is likely affecting a regulatory gene which either directly or indirectly influences the genes involved in monoterpene, sesquiterpene and FPC biosynthesis.

There are three major types of genetic regulation; transcriptional, translational and epigenetic regulation. Broun *et al*. [45] suggests that translational and epigenetic regulation are more important in primary metabolism, whereas transcriptional regulation is the dominant mode of controlling secondary metabolism and this seems to be the case for terpene biosynthesis. To date, there is little evidence for the role of translational and epigenetic regulation on terpene biosynthesis. There are two examples of translational regulation from sesquiterpene production in *Arabidopsis thaliana* flowers [46] and isoprene production in the leaves in poplar (*Populus canescens*) [47]. The only example of epigenetic regulation within the terpene biosynthetic pathway comes from the Madagascar Periwinkle (*Catharanthus roseus*), where the phosphorylation of the transcription factor WRKY8 affects HMGR activity, which in turn affects total terpene yield [48].

Six transcription factors have been identified and confirmed to influence terpene biosynthesis. These are a WRKY from cotton (*Gossypium arboretum*) [49]; an ORCA and a WRKY from Madagascar Periwinkle (*Catharanthus roseus*) [48, 50]; and a WRKY and two ERFs from sagewort (*Artemisia annua*) [51–52]. With the exception of one (the WRKY from Madagascar Periwinkle—[48]), all of these transcription factors act directly on terpene synthases [49–52].

We identified 24 transcription factors differentially expressed between the leaves of the two branches in each species (Table 7). Of these, seven are common across the two species, with just one showing the opposite pattern of up-regulation in the two species. We identified transcription factors belonging to 12 different families differentially expressed between the resistant and susceptible leaves in the two species. The serum response factor (SRF) transcription factor family has the greatest number of transcripts differentially expressed between the leaves of the two branches in *E*. *melliodora* and the WRKY transcription factor family has the greatest number of transcripts differentially expressed between the leaves of the two branches in *E*. *sideroxylon* (Table 7).

**Table 7:** Transcription factors significantly differentially expressed in the leaves of both mosaic trees.

*E*. *melliodora*				*E*. *sideroxylon*			
LG	Domain	logFC	Gene Name	Gene Name	logFC	Domain	LG
7	B3	0.79	Eucgr.G02345	Eucgr.G02345	1.28	B3	7
8	bZIP	1.12	Eucgr.H04338	Eucgr.H04338	0.98	bZIP	8
9	bZIP	-0.31	Eucgr.I01289	Eucgr.I01289	-0.54	bZIP	9
3	CBF	0.40	Eucgr.C00842	Eucgr.C00842	1.07	CBF	3
4	HEX	0.56	Eucgr.D02105	Eucgr.D02105	1.30	HEX	4
11	HEX	0.55	Eucgr.K02448	Eucgr.K02448	0.65	HEX	11
8	bZIP	0.96	Eucgr.H04038	Eucgr.H04038	-2.82	bZIP	8
1	AP2	1.02	Eucgr.A02827	Eucgr.C00664	-1.07	HSF	3
1	AP2	0.96	Eucgr.A02834	Eucgr.E00555	-0.75	HSF	5
3	AP2	-0.82	Eucgr.C00358	Eucgr.A02338	-1.21	HSF	1
6	AP2	-0.46	Eucgr.F01164	Eucgr.I00929	-0.99	HSF	9
9	AP2	-0.37	Eucgr.I00422	Eucgr.K00238	-1.54	HSF	11
12	CBF	0.40	Eucgr.L00153	Eucgr.B03006	-1.47	MYB	2
1	GRAS	-0.66	Eucgr.A01051	Eucgr.F00002	-0.65	MYB	6
1	GRAS	-0.38	Eucgr.A01279	Eucgr.A01857	0.95	MYB	1
1	HEX	0.45	Eucgr.A01715	Eucgr.D00972	-0.84	MYB	4
3	MYB	6.82	Eucgr.C03026	Eucgr.J02073	-0.80	MYB	10
7	NAM	1.10	Eucgr.G01758	Eucgr.K01255	0.75	Sigma	11
2	SRF	0.34	Eucgr.B00522	Eucgr.B03520	-1.73	WRKY	2
11	SRF	0.46	Eucgr.K00197	Eucgr.A01990	-1.87	WRKY	1
11	SRF	0.33	Eucgr.K00198	Eucgr.E04011	-3.08	WRKY	5
11	SRF	0.50	Eucgr.K00199	Eucgr.C00077	-1.15	WRKY	3
11	SRF	0.43	Eucgr.K00201	Eucgr.F00187	-1.07	WRKY	6
11	SRF	0.43	Eucgr.K00208	Eucgr.F03955	-0.77	WRKY	6

Genes above the horizontal line are significantly differentially regulated in both species, and those below the line are only significantly differentially regulated in one species. LG refers to the linkage group on which the gene is found in the *E*. *grandis* genome (Joint-Genome-Institute 2006–2013), binding domain denotes which binding domains are found in the sequence and therefore which transcription factor family each gene belongs to (Joint-Genome-Institute 2006–2013); the logFC column contains log fold change values, with a positive number representing an over-abundance of this transcript in the leaves of the resistant branch, and a negative number representing an over-abundance of this transcript in the leaves of the susceptible branch; gene name shows the name of the gene the transcript matches, using the *Eucalyptus grandis* genome annotations in phytozome (Joint-Genome-Institute 2006–2013). The transcription factor families are B3: B3 DNA binding domain; bZIP: basic leucine zipper domain; CBF: C-repeat binding factor; HEX: hematopoietically-expressed homeobox protein; AP2: activating protein 2; GRAS: gibberellic-acid insensitive (GAI), repressor of GAI (RGA) and SCARECROW (SCR); MYB: MYB proto-oncogene protein; NAM: No apical meristem protein; SRF: serum response factor; HSF: heat shock factor; Sigma: Sigma factor, a plastidal RNA polymerase cofactor; WRKY: W-box binding domain containing amino acid sequence WRKY.

Transcription factors containing the MYB and WRKY binding domains have previously been shown to interact directly with terpene synthases [51, 53], and with other genes involved in plant secondary metabolite biosynthesis [54–55]. Therefore, we predict transcription factors with these binding domains are involved in the development and maintenance of phenotypic mosaicism. Both of these types of transcription factors are differentially expressed between the leaves of the two branches in the *E*. *sideroxylon* mosaic (there is only one MYB and no WRKYs differentially expressed in the *E*. *melliodora* mosaic—Table 7), suggesting they may play an important role in the phenotypic mosaicism of *E*. *sideroxylon*.

There is a higher concentration of sesquiterpenes in the susceptible chemotype of each mosaic tree [28] and since sesquiterpenes are produced by a pathway containing eight genes with up to six copies in some eucalypts, we hypothesise that there will be either an up-regulation of promoter-type transcription factors in the leaves of the susceptible branch, or a down-regulation of repressor-type transcription factors in the leaves of the susceptible branch. Nonetheless, it is possible to identify candidate transcription factors that may play an important role in the development and maintenance of mosaicism in these eucalypts. Henery *et al*. [44] identified QTLs in the *E*. *nitens* associated with foliar monoterpene, sesquiterpene and FPC concentrations. There was a large clustering of QTLs on linkage groups 7, 8 and 9; with the latter two containing QTLs associated with all three groups of compounds [44]. In each mosaic there are four transcription factors differentially expressed between the leaves of the resistant branch and those of the susceptible branch that are found on these linkage groups. Three of these transcription factors are differentially regulated in the same direction in the two species, including the bZIP, and B3 families (Table 7). The remaining two transcription factors are both up-regulated in the leaves of the susceptible branch, and belong to the AP2 and HSF families in *E*. *melliodora* and *E*. *sideroxylon*, respectively (Table 7). Any of these five transcription factors could be responsible for controlling mosaicism in these trees and we will adopt a transgenic approach in the future with a model species to test the role of these transcription factors.

### Comparison to previous study

In this study we generated a much larger sequence dataset (193,877,527 reads from 10 samples) than in the previous study (277,725 reads from two samples) [29]. As a result we were able to use a more robust and detailed statistical approach in this study. We found the up-regulation of GO categories in the leaves of the resistant or susceptible branches of the *E*. *melliodora* to be the same using both data sets. We previously found four methylerythritol phosphate (MEP) pathway genes are up-regulated in the resistant branch of the *E*. *melliodora* mosaic [29]. We also found that the mevalonate (MVA) pathway genes were expressed at the same level in the leaves of both chemotypes of this mosaic [29]. However, in this study we show that the same gene in the MEP and different copies of the same gene in the MVA pathway are differentially regulated between the two chemotypes in both mosaics. Since we were able to include more samples and therefore use more robust statistical analyses in this study, we were also able to show that entire pathways are differentially regulated and we had sufficient data to investigate the differential expression of individual genes within a pathway (the terpene biosynthetic genes). Despite the similar findings in these two studies, there are several crucial differences that show we have made a substantial improvement to our understanding of somatic mutations in long-lived trees.

### Future Directions

In the future we will investigate additional phenotypic variation guided by genetic differences. For example, the pathway analysis suggests that genes involved in the synthesis of flavonoids, carotenoids, betains, wax and sulphur-containing compounds are all differentially expressed between the chemotypes of the *E*. *melliodora* mosaics and those involved in the synthesis of betains, wax, glucosinolates, sulphur-containing compounds and some of the flavonoids are differentially regulated between the chemotypes of the *E*. *sideroxylon* mosaic, which points towards their being phenotypic differences in the products of these pathways. We will measure these plant secondary metabolites to confirm whether we can predict phenotypic differences from gene expression patterns. Additionally we are undertaking experiments to determine the function of all of the expressed terpene synthases in the leaves of the two mosaic trees. There is some evidence to suggest that only a few of these (up to 30) will be functional and contributing to the foliar terpene profile. Finally, we are investigating the function of the transcription factors identified in this study, to determine if these do play a role in the development or maintenance of chemical mosaicism.

We found clear differences in gene expression and pathway regulation between two chemotypes of two *Eucalyptus* mosaic trees. Patterns in the terpene biosynthetic genes are consistent with foliar terpene differences between the chemotypes and pathway regulation patterns point towards a number of other metabolites showing differences between the leaves of each chemotype. This suggests that a similar approach could be used to predict what metabolites vary within a single individual. We identified a list of transcription factors that are good candidates for the development and maintenance of phenotypic mosaicism in these species of *Eucalyptus*.

## Materials and Methods

### Plant material

Foliage samples were collected at two sites in south-eastern NSW, Australia at Yeoval (32°45’10”S 148°39’20”E—*E*. *melliodora*) and at Cumnock (32°57’55”S 148°46’44”E—*E*. *sideroxylon*) known to contain individual trees of *Eucalyptus melliodora* and *Eucalyptus sideroxylon* previously identified as foliar chemical mosaics [25–26]. Using a truck-mounted elevated platform, five samples of fully expanded leaves were collected from both chemotypes of both mosaic trees, each from a separate branch. The leaves were immediately stripped from the branch and put into labelled envelopes and quickly immersed in liquid nitrogen. They were subsequently stored at -80°C until further use.

### Ethics statement

Institutional Ethics Committee Approval was not required for this study. This study was undertaken on private land. Please contact the corresponding author for further details.

### RNA extraction and library preparation

The leaves were ground to a fine powder using a mortar and pestle, under liquid nitrogen. We extracted total RNA from the leaf powder using the Spectrum Total RNA Kit as per the manufacturer’s instructions (Sigma Aldrich, MO). The quality and quantity of the RNA was assessed using gel electrophoresis (1x TAE, 1% agarose and ethidium bromide), a Nanodrop ND-1000 Spectrophotometer (ThermoFisher Scientific, Vic) and a Bioanalyzer 2100 (Agilent Technologies, CA).

We aimed to sequence the transcriptome of three samples from both chemotypes of each mosaic, however the sequencing of two samples of the resistant chemotype of the *E*. *sideroxylon* failed. Therefore we have data from three samples of resistant and susceptible *E*. *melliodora*, three samples of susceptible and one sample of resistant *E*. *sideroxylon*.

We then used the Illumina TruSeq RNA library preparation kit as per manufacturer’s instructions (Illumina Inc., CA). The libraries were validated on a Bioanalyzer 2100 (Agilent Technologies, CA), pooled and sequenced on an Illumina HiSeq 2000 at the Biomolecular Research Facility at the Australian National University, using the 100 bp paired-end run.

### Data Quality Assurance

Raw reads from the Illumina HiSeq 2000 sequencer were visually assessed for their quality using FastQC [56]. Quality value distribution along the length of tags was acceptable (median value >30). Bad quality reads with a mean Phred-like score <20 were removed using Trimmomatic [57]. Reads were also trimmed at either ends for poor quality end-regions using Trimmomatic such that mean quality in last 4nt window was >20. All paired reads which had at least 50 nt on both reads of the pair were retained for further analyses.

### Data Analysis

The genome sequence of *E*. *grandis* (8X mapped *BRASUZ1*) was obtained from the Joint Genome Institute’s Phytozome resource [58–59], along with associated gene annotation files. All reads were aligned to the *E*. *grandis* genome reference sequence using TopHat (version 2.0.4) with default parameters. Aligned BAM files were further processed using Cufflinks [60] (version 2.0.0, default parameters) to identify and assemble known and novel transcripts. Then Cuffmerge [60] was used to produce non-redundant set of known and novel transcripts for our dataset. Read alignments were overlapped with gene annotations for *E*. *grandis* using intersectBed [61] (BedTools version 2.16.2) and sequenced fragment counts were then generated for each gene in each sample using custom Perl scripts. Novel transcripts were not further analysed. All custom Perl and R scripts used in the execution of this research are available on request.

Differential expression analysis for genes between samples was performed using the edgeR package (version 2.6.12) [62] following the author’s recommendations. Briefly, the sum of mapped tag counts for each sample was used as its effective library size. Read counts per gene were first normalized for library size to obtain counts per million mapped tags. These counts were further normalized for total RNA output using the TMM method [63]. We selected for further analysis all genes that contributed to the top 95% of the cumulative counts. Zero tag counts were adjusted to the mean value of the lowest normalized count and zero. For each groups of samples, dispersion estimates were obtained using the quantile-adjusted conditional maximum likelihood (qCML) method. Subsequently, differential expression tests were computed for each gene and p-values were corrected for multiple-testing using the Benjamini-Hochberg procedure to its False Discovery Rate (FDR). Genes were identified as differentially expressed if the p-value and FDR for differential expression was ≤0.05 and the mean expression count for the gene was at least 2 on a log2 counts per million scale across all samples. This count refers to at least 28 tag counts.

Pathway analysis was performed in Mapman [64] using the corresponding *Arabidopsis thaliana* gene identifiers (AT numbers) for each *Eucalyptus grandis* gene identifier (Eugra number) and log fold change data. The AT numbers corresponding to *Eucalyptus* genes were obtained from annotations in the Phytozome database using custom Perl scripts. Duplicate AT numbers were removed, and we used default parameters to map the transcripts using the AGI database of *Arabidopsis thaliana* (Ath_AGI_TAIR9_Jan2010 in Mapman).

Gene Ontology (GO) term enrichment analysis was performed using g:Profiler [65–66] using *Arabidopsis thaliana* gene identifiers corresponding to *Eucalyptus* genes. We ordered up- or down-regulated genes according to their fold change values and opted for the *“sorted query”* option in g:Profiler against the background of all genes that we had identified as being expressed in our samples.

### Terpene synthase amplification

All transcripts were amplified using a two-step cDNA synthesis protocol. For the first step, synthesis of the first complimentary DNA strand, we used Moloney Murine Leukemia Virus (H^-^ Point Mutant) Reverse Transcriptase (M-MLV H^-^ RT—Promega Corporation, WI) and a poly-T primer following the manufacturers’ instructions. From here we could use gene specific primers to amplify terpene synthases. We used sequence data from the transcriptome to design full-length primers to amplify terpene synthases. We chose sequences for which we had good quality data over the start and stop codons, as well as over at least two of the conserved domains (RRX_8_W, RLLRQ, RWW, DDXXD or NTE/DSE). There were eight sequences that matched these criteria. Each terpene synthase was amplified from 20–65 ng of this first strand cDNA in 25 μl reactions containing 0.625 units of GO Taq and a final concentration of 1x GO Taq buffer (Promega Corporation, WI), 200 μM dNTPs, 800 nM forward primer and 800 nM reverse primer. We cloned these amplicons into the sequencing vector (pGEM-T easy—Promega Corporation, WI) and obtained good quality sequence from each.

In each case the quality and quantity of the amplicons was assessed using gel electrophoresis (1x TAE, 1% agarose and ethidium bromide) and a Nanodrop ND-1000 Spectrophotometer (ThermoFisher Scientific, Vic), respectively. Amplicons were sequenced on an ABI 3130 Genetic Analyzer (Applied Biosystems, CA), following manufacturer’s instructions using BigDye 3.1.

### Functional characterisation of terpene synthases

We used the Champion pET Directional TOPO Expression Kit (specifically pET200D —Life Technologies, NY) as per the manufacturers’ instructions with one exception. The plasmids positive for the insert were harvested from the TOP10 cells and sent to Halle (Saale), Germany where they were transformed into the BL21 Star (DE3) cells and gene over-expression was induced with IPTG (isopropyl β-D-1-thiogalactopyranoside). After expression, the enzymes were extracted and incubated with geranyl pyrophosphate (GPP), the precursor to all monoterpenes, and farnesyl pyrophosphate (FPP), the precursor to all sesquiterpenes [67]. The volatile products were collected using a solid phase micro-extraction (SPME) method and analysed by gas chromatography-mass spectrometry [68].
